# Supplementary material for: Gene Expression in Muscle-Invasive and Non-Muscle-Invasive Bladder Cancer Cells Exposed to Hypoxia
Source: Cancers (Basel). 2025 Aug 11;17(16):2624. doi: 10.3390/cancers17162624 (PMC12384837; doi:10.3390/cancers17162624)
Supplement: Supplementary file 1 [file cancers-17-02624-s001.zip › cancers-3752987-supplementary.pdf]

Supplementary data

Research gap and study uniqueness

Search Strategy

The research strategy involved iterative refinement, with additional keywords and Boolean operators (AND, OR) incorporated to insure comprehensive literature coverage. The search was limited to English-language studies and the reference lists of relevant articles were examined, along with the citation to further expand it.

Study Selection Criteria

The inclusion criteria for this review were:

- Studies focusing on the gene expression in bladder cancer only.
- Studies examining the expression of gene and hypoxia.
- Studies examining the response to hypoxia and gene signature in bladder cancer.
- Studies published in English.

The exclusion criteria were:

- Studies that focus on bladder cancer only without hypoxia/gene expression.
- Studies that were not related to hypoxia/gene expression.
- Studies not published in English.

Addressing a literature

Literature research on gene expression differences in bladder cancer under hypoxia, specifically comparing NMIBC and MIBC, was performed. The search was divided into two categories: patient studies and cell line studies. The resulting studies were assessed.

Significance of the evidence

This systematic review confirmed that no previous study has assessed gene signatures in both MIBC and NMIBC cells under multiple hypoxic levels, highlighting the novelty of the study design.The novelty of our study is that we compared the expression of genes for 6 high grade MIBC (Grade 3) to two low grade NMIBC (grade 1 and 2). Additionally, we identified and compared the number of genes expressed and pathways for those genes to identify a disease progression and a prognostic/predictive markers.

Supplementary Table S1

	RT4	RT112	HT1376	T24	UMUC-3	J82
	P4HA2	P4HA2	0	P4HA2	P4HA2	P4HA2
	DPYSL2	DPYSL2	DPYSL2	DPYSL2	DPYSL2	DPYSL2
	FUT11	FUT11	FUT11	FUT11	FUT11	FUT11
	0	RSBN1	RSBN1	RSBN1	0	RSBN1
	SAV1	0	0	SAV1	SAV1	0
	SYDE1	SYDE1	SYDE1	SYDE1	SYDE1	SYDE1
	0	0	0	CAV1	0	0
	PDLIM2	0	PDLIM2	PDLIM2	0	0
	LRP1	0	0	LRP1	0	0
	0	ITGA5	0	0	ITGA5	0
	0	AHNAK2	0	AHNAK2	AHNAK2	0
	0	0	0	0	COL5A1	0
	0	0	0	0	0	0
	0	0	0	0	0	0
	0	0	0	0	0	0
	0	0	0	0	0	SLC16A1
	0	0	0	GULP1	GULP1	0
	0	0	0	0	0	0
	SRPX	0	0	0	0	0
	0	0	0	0	0	0
	0	0	0	0	DAAM1	0
1%	0	0	0	0	0	0
	P4HA2	P4HA2	P4HA2	P4HA2	P4HA2	P4HA2
	DPYSL2	DPYSL2	DPYSL2	DPYSL2	DPYSL2	DPYSL2
	FUT11	FUT11	FUT11	FUT11	FUT11	FUT11
	0	SLC2A3	SLC2A3	SLC2A3	0	SLC2A3
	SAV1	SAV1	SAV1	SAV1	SAV1	0
	SYDE1	SYDE1	SYDE1	SYDE1	SYDE1	SYDE1
	0	CAV1	CAV1	CAV1	0	0
	PDLIM2	0	PDLIM2	PDLIM2	0	0
	LRP1	0	0	LRP1	LRP1	0
	0	ITGA5	0	0	0	0
	0	AHNAK2	0	AHNAK2	AHNAK2	0
	0	0	0	0	COL5A1	0
	0	0	TRAM2	0	0	0
	0	0	0	0	0	0
	0	0	0	0	0	0
	0	0	0	0	0	0
	0	0	0	GULP1	0	0
	0	0	0	0	0	0
	SRPX	0	0	0	0	0
	0	0	0	0	0	0
	0	0	0	0	0	0
0.2%	0	0	0	0	0	0

	P4HA2	P4HA2	P4HA2	P4HA2	P4HA2	P4HA2
	DPYSL2	DPYSL2	DPYSL2	DPYSL2	DPYSL2	DPYSL2
	FUT11	FUT11	FUT11	FUT11	FUT11	FUT11
	SLC2A3	0	SLC2A3	SLC2A3	0	SLC2A3
	SAV1	SAV1	SAV1	SAV1	0	0
	SYDE1	SYDE1	SYDE1	SYDE1	SYDE1	SYDE1
	CAV1	CAV1	CAV1	CAV1	0	0
	0	PDLIM2	0	0	PDLIM2	PDLIM2
	LRP1	LRP1	0	LRP1	LRP1	0
	ITGA5	ITGA5	0	0	ITGA5	0
	AHNAK2	0	0	AHNAK2	AHNAK2	0
	0	0	0	0	COL5A1	0
	0	0	TRAM2	TRAM2	0	0
	0	CAD	0	0	CAD	CAD
	0	0	0	0	0	0
	0	0	0	0	0	0
	0	0	0	GULP1	0	0
	DSC2	0	0	0	0	0
	0	SRPX	0	0	0	0
	0	0	0	0	0	0
	0	0	0	0	0	0
0.1%	0	0	0	0	0	0
Genes in 24 gene signature but not expressed in bladder cancer cells						
CYP1B1	-	-	-	-	-	-
GLG1	-	-	-	-	-	-

Supplementary Table S2. Genes up-regulated in at least 2 MIBC but not NMIBC cancer cells exposed to (A) 0.1% (B) 0.2% or (C) 1% O_2 for 24h.

(A)

ACAP1	*BHLHE41*	*DUSP1*	*KDM7A*	*NRN1*	*PPP1R3G*	*SEC61G*	*TNFAIP3*
ADAMTS16	*BIRC3*	*ERRFI1*	*KLF7*	*PDK1*	*PRKAR2B*	*SEMA7A*	*TNIP1*
ADARB1	*C1orf21*	*ETS1*	*L1CAM*	*PFKL*	*PROSER2*	*SLC2A1*	*TNNT1*
ADSS1	*C4orf47*	*F3*	*LNPK*	*PGAM1*	*PRR5L*	*SPNS2*	*TXNIP*
AHNAK2	*CA12*	*FAM117B*	*LOX*	*PGAM4*	*RAP1GAP2*	*SPRY1*	*UPRT*
AKAP12	*CAMK2N1*	*FAM83A*	*MLLT3*	*PIAS2*	*RAPGEF1*	*SPRY3*	*VEGFA*
ALDOA	*CCP110*	*FGF11*	*MME*	*PKIA*	*RASSF2*	*SSBP2*	*WSB1*
ALKBH5	*CDK19*	*GPR160*	*MTUS1*	*PLEKHA2*	*RBPJ*	*STC1*	

ANGPTL4	*CRLF2*	*GYS1*	*MUC1*	*PLIN2*	*RHOB*	*TCP11L2*	
ARFGEF3	*CSPG5*	*HK2*	*NAV1*	*PPFIA4*	*RNF122*	*TGM2*	
ARHGAP31	*DIPK2A*	*IGF2*	*NEDD4L*	*PPP1R3B*	*RNF185*	*TMEM191B*	
ARRDC4	*DTNA*	*IGFBP3*	*NFIL3*	*PPP1R3C*	*SAP30*	*TMEM191C*	

(B)

ADARB1	*BHLHE41*	*DYRK1B*	*HILPDA*	*MRPL23*	*PGM1*	*RORA*	*TNNT1*
ADSS1	*BIRC3*	*EGLN1*	*HK2*	*MUC1*	*PIAS2*	*SAP30*	*TXNIP*
AHNAK2	*C1orf21*	*ERO1A*	*IGF2*	*MYO1E*	*PLEKHA2*	*SLC2A1*	*VEGFA*
AKAP12	*C4orf47*	*ERRFI1*	*IGFBP3*	*NAV1*	*PLIN2*	*SNX33*	*WSB1*
ALDOA	*C8orf58*	*F3*	*KLF7*	*NFIL3*	*PNRC1*	*SPNS2*	*YEATS2*
ANKRD37	*CA12*	*FAM117B*	*L1CAM*	*NREP*	*PPP1R3B*	*SPRY3*	*ZNF175*
ARFGEF3	*CRLF2*	*FGF11*	*LGALS8*	*PDK3*	*PPP1R3C*	*STC1*	
ARRDC3	*DTNA*	*GBE1*	*LOX*	*PDLIM2*	*PPP1R3G*	*TCP11L2*	
ARRDC4	*DUSP1*	*GPR160*	*LRP1*	*PFKL*	*RNF122*	*TGM2*	
BHLHE40	*DYNC2I1*	*GYS1*	*MLLT3*	*PGAM1*	*RNF24*	*TMEM45A*	

(C)

ANKRD37	*C4orf3*	*MXI1*	*PLAC8*	*RORA*	*SLC2A3*	*TCAF2*	
ARRDC3	*FAM162A*	*PFKFB3*	*PLEKHA2*	*SFXN3*	*SPAG4*	*TXNIP*	
BHLHE40	*MRPL23*	*PGK1*	*PPP1R3G*	*SLC2A14*	*STC1*	*VEGFA*	

Supplementary Table S3. Genes up-regulated in both NMIBC cancer cells but not in at least 2 MIBC cancer cells exposed to (A) 0.1% (B) 0.2% or (C) 1% O_2 for 24h.

(A)

ACER2	*CMC4*	*GOLGA8A*	*ISG20*	*MKNK2*	*PDGFB*	*PTPRR*	*UPK1A*
AOX1	*DEPTOR*	*GOLGA8B*	*ITGA5*	*MT1F*	*PDK1*	*RIMKLA*	*YPEL2*
ARID3A	*DERA*	*HAS3*	*KLF11*	*MT1X*	*PDLIM2*	*RRAGD*	
B3GALT4	*DTNB*	*HEY1*	*LBH*	*MT2A*	*PGM1*	*SAMD4A*	
C8orf58	*EEF1A2*	*IGFBP6*	*LGALS1*	*MXD1*	*PODXL*	*SERPINE1*	
CAVIN1	*FBXO32*	*IL1R2*	*LPIN3*	*MYO5B*	*PRDM1*	*SH3BP2*	
CITED2	*GDE1*	*IL36RN*	*MAF*	*NREP*	*PRELID2*	*SPOCK1*	
CLEC2B	*GJA1*	*IPMK*	*MB*	*PCOLCE2*	*PRKAA2*	*STC2*	

(B)

CLEC2B	*FBXO32*	*HEY1*	*LBH*	*MB*	*PCED1B*	*PRKAA2*	*STC2*
CMC4	*GJA1*	*IL1R2*	*LGALS1*	*MOB3A*	*PGM2*	*SERPINE1*	*TMEM45A*
DEPTOR	*HAS3*	*ITGA5*	*MAF*	*MT2A*	*PRELID2*	*SH3BP2*	*WDR54*

(C)

ALDOC	*CSRP2*	*GJA1*	*LGALS1*	*PGF*	*PLOD2*	*SERPINE1*	
CA9	*DEPTOR*	*LBH*	*PAM*	*PGM1*	*PNRC1*		

Supplementary Figure S1. Median gene expression values for 77 (76) and 5 gene panels identified as prognostic in TCGA cohort in pimonidazole low and high stained regions in a bladder cancer cell derived xenograft. * P<0.05; **p<0.01

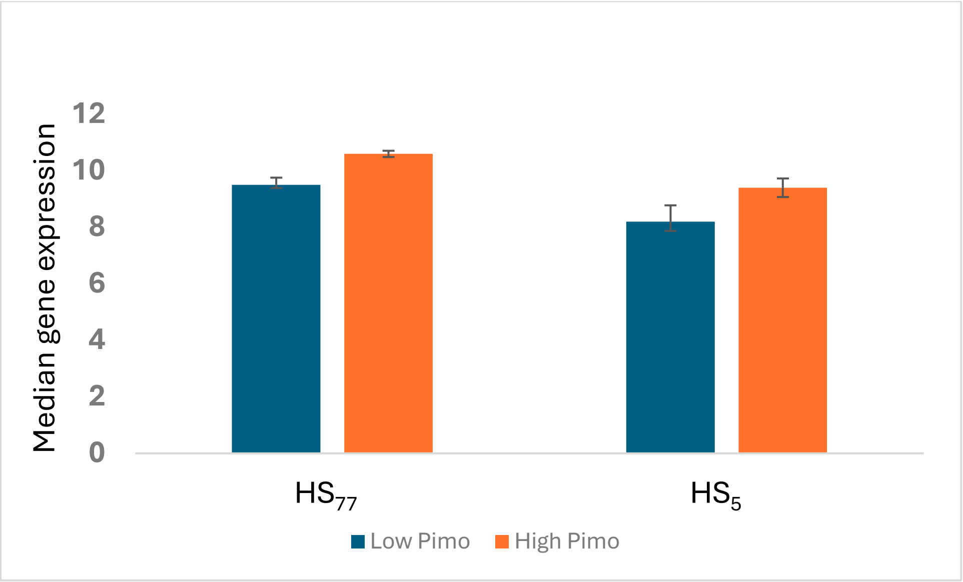

Supplementary Figure S2. Overall survival curves for patients with bladder cancer stratified by median expression of the 77genes (ME_{77}) upregulated at least 3 bladder cancer cell lines or in 5 genes common to the ME_{77} and Yang 24 gene bladder cancer hypoxia signature respectively in BCON (A and B), GSE38164 (C and D), R19915 GLP8333 (E and F), R19915 GLP5186 (G and H), and IMvigo210 (I and J).

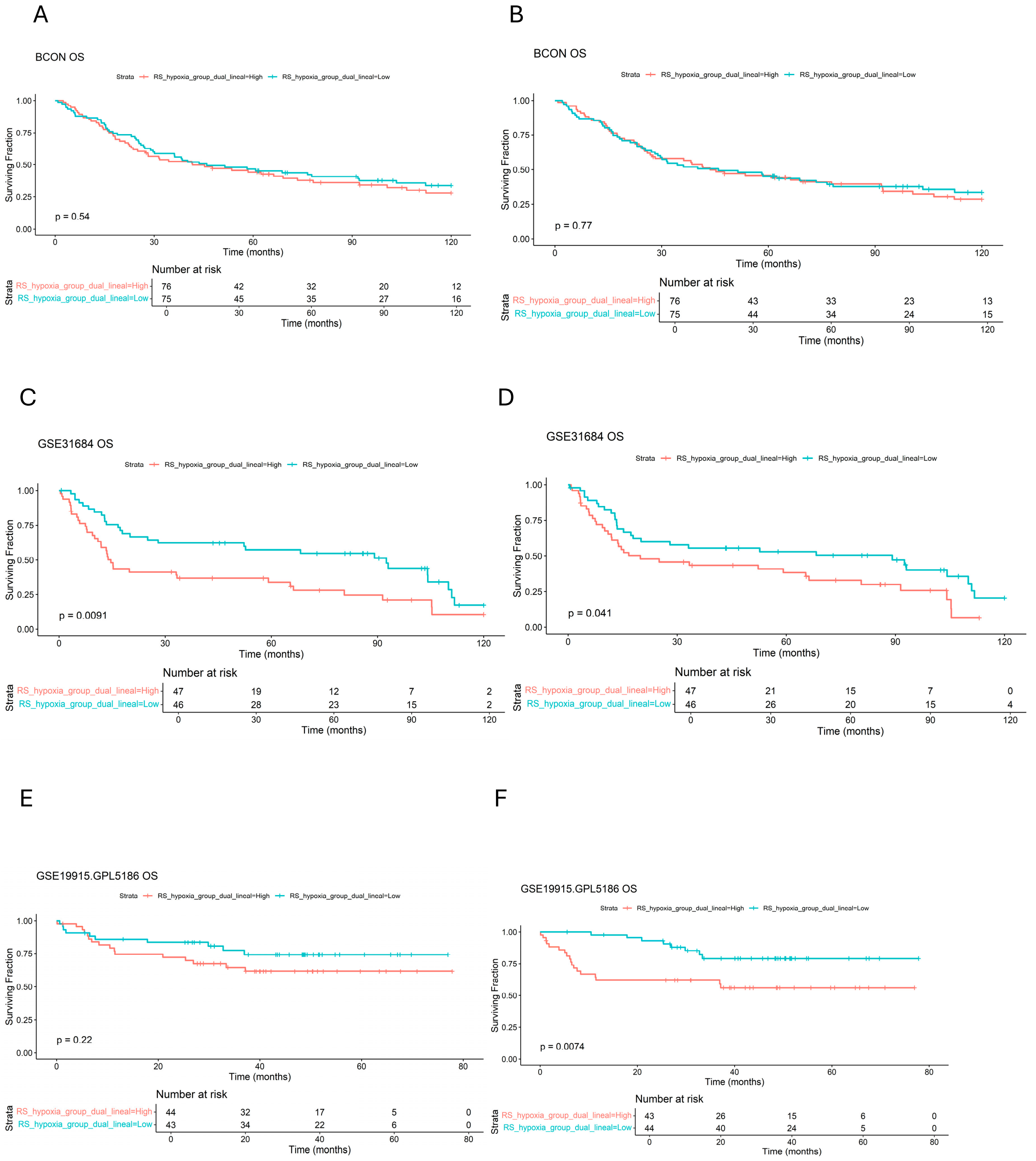

G

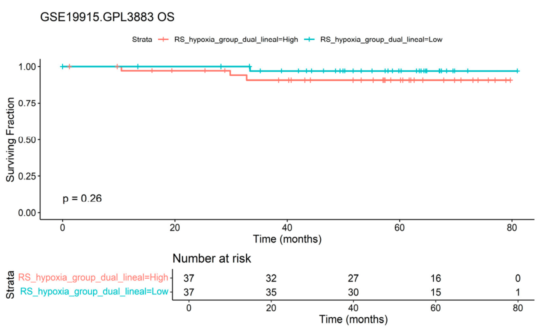
GSE19915.GPL3883 OS
Strata
RS_hypoxia_group_dual_lineal=High
RS_hypoxia_group_dual_lineal=Low
Surviving Fraction
1.00
0.75
0.50
0.25
0.00
p = 0.26
0
20
40
60
80
Time (months)
Number at risk
Strata
RS_hypoxia_group_dual_lineal=High 37 32 27 16 0
RS_hypoxia_group_dual_lineal=Low 37 35 30 15 1
0
20
40
60
80
Time (months)

H

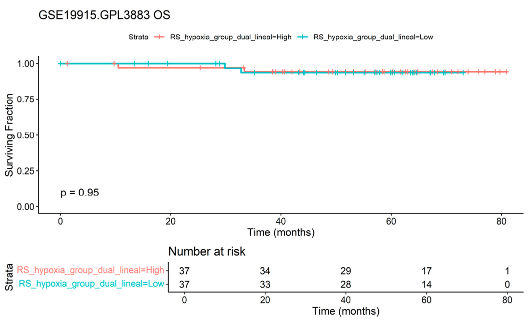
GSE19915.GPL3883 OS
Strata
RS_hypoxia_group_dual_lineal=High
RS_hypoxia_group_dual_lineal=Low
Surviving Fraction
1.00
0.75
0.50
0.25
0.00
p = 0.95
0
20
40
60
80
Time (months)
Number at risk
Strata
RS_hypoxia_group_dual_lineal=High 37 34 29 17 1
RS_hypoxia_group_dual_lineal=Low 37 33 28 14 0
0
20
40
60
80
Time (months)

I

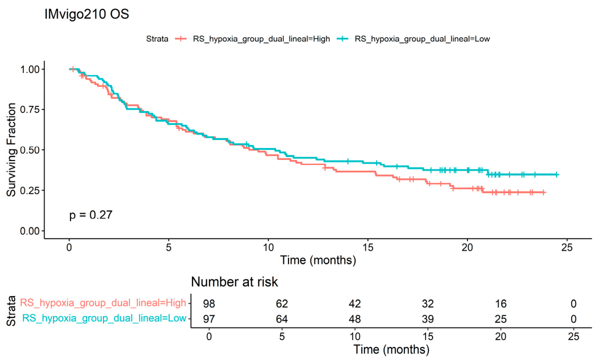
IMvigo210 OS
Strata
RS_hypoxia_group_dual_lineal=High
RS_hypoxia_group_dual_lineal=Low
Surviving Fraction
1.00
0.75
0.50
0.25
0.00
p = 0.27
0
5
10
15
20
25
Time (months)
Number at risk
Strata
RS_hypoxia_group_dual_lineal=High 98 62 42 32 16 0
RS_hypoxia_group_dual_lineal=Low 97 64 48 39 25 0
0
5
10
15
20
25
Time (months)

J

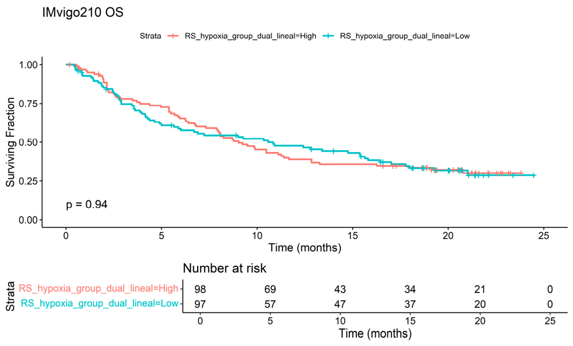
IMvigo210 OS
Strata
RS_hypoxia_group_dual_lineal=High
RS_hypoxia_group_dual_lineal=Low
Surviving Fraction
1.00
0.75
0.50
0.25
0.00
p = 0.94
0
5
10
15
20
25
Time (months)
Number at risk
Strata
RS_hypoxia_group_dual_lineal=High 98 69 43 34 21 0
RS_hypoxia_group_dual_lineal=Low 97 57 47 37 20 0
0
5
10
15
20
25
Time (months)
